# The changing faces of migraine

**DOI:** 10.1186/s10194-019-1006-z

**Published:** 2019-05-10

**Authors:** Paolo Martelletti, Messoud Ashina, Lars Edvinsson

**Affiliations:** 1 Department of Clinical and Molecular Medicine, Sapienza University, Rome, Italy; 2 Department of Neurology, Copenhagen University, Copenhagen, Denmark; 3 Department of Clinical Sciences, Lund University, Lund, Sweden

Migraine represents today the crossroads of several scientific evidences, such as lofty planetary prevalence, serious disability, damaged quality of life, reduced productive capacity, costly healthcare resulting in a creeping sentiment of social stigma [1–3]. From the same crossroads, the migraine paradigm as a multimorbid disease flows out [4]: perhaps multi-systemic, however to be scanned and evaluated at a distance as the horizon, in order to avoid the risk of parcelling its real extent.

From before the triptans era, in less than 30 years we have passed from the common acceptance of migraine as an ineluctable family heredity to the evidence of a complex genetic trait involving genomics, metabolomics and, perhaps in the near future, also proteomics [5, 6].

In the meantime, the awareness of migraine as real disease has been widespread in the general population and even physicians’ education on migraine has made giant steps forward at different levels of research, professionalism and safe prescription [7–10].

Now that we are facing a new era, the Calcitonin Gene Related Peptide (CGRP) one, with new perspectives and hopes that only a new pharmacological class dedicated to the prevention of migraine can offer, we must not forget what has made this possible: solid and undisputable scientific evidences acquired over the years first on animal and then on human models [11, 12].

This topical collection helps us to orient the readership in the great mass of data that emerge from literature and systematize them in a series of comprehensive reviews, progressively centered on chained topics. From the classic burden of disease to the new acquisitions of genetics, from the new pathophysiology CGRP-centered of migraine to the description of the various new molecules and their correct and safe therapeutic application, up to the rocks of refractoriness and the unavoidable ethical and economic need to identify the right patient for the right drug using the precision medicine [13, 14].

Satisfied all the parameters, before the end of this path we could have the chance to mark out the vanishing point of migraine and to score the greatest try to be devoted to each migraine patient.
